# Trajectories and correlates of mental health among urban, school-age children during the COVID-19 pandemic: a longitudinal study

**DOI:** 10.1186/s13034-024-00712-4

**Published:** 2024-03-14

**Authors:** Rachel Oblath, Rohan Dayal, J. Krystel Loubeau, Julia Lejeune, Jennifer Sikov, Meera Savage, Catalina Posse, Sonal Jain, Nicole Zolli, Tithi D. Baul, Valeria Ladino, Chelsea Ji, Jessica Kabrt, Lillian Sidky, Megan Rabin, Do Yoon Kim, Imme Kobayashi, J. Michael Murphy, Arvin Garg, Andrea E. Spencer

**Affiliations:** 1 Department of Psychiatry, Boston University Chobanian & Avedisian School of Medicine, Boston Medical Center, Boston, MA USA; 2 BEST Partnership for Behavioral Health, Racial, and Social Justice, Boston, MA USA; 3 Department of Psychiatry, Boston Medical Center, Boston, MA USA; 4 School of Public Health, Boston University, Boston, MA USA; 5 Department of Psychology, University of Illinois at Chicago, Chicago, IL USA; 6 Department of Psychiatry & Behavioral Sciences, Duke University School of Medicine, Durham, NC USA; 7 Department of Psychiatry, Massachusetts General Hospital, Boston, MA USA; 8 Harvard Medical School, Boston, MA USA; 9 Department of Pediatrics, Child Health Equity Center, University of Massachusetts Chan Medical School, Worcester, MA USA; 10 University of Massachusetts Memorial Children’s Medical Center, Worcester, MA USA; 11 Pritzker Department of Psychiatry and Behavioral Health, Ann & Robert H. Lurie Children’s Hospital of Chicago, 225 E Chicago Ave, Chicago, IL 60611 USA; 12 Department of Psychiatry and Behavioral Sciences, Northwestern University Feinberg School of Medicine, IL Chicago, USA; 13 Department of Psychology, College of Arts, Sciences, and Education, Florida International University, FL Miami, USA; 14 New York University Grossman School of Medicine, NY New York City, USA

**Keywords:** Child mental health, Social risk factors, Minority health, COVID-19

## Abstract

**Background:**

The COVID-19 pandemic posed numerous obstacles to psychosocial wellbeing for children. We conducted a longitudinal study to evaluate child mental health and social risks during the pandemic.

**Methods:**

Participants were 172 caregivers of children aged 6–11 years old who attended well child visits within 6 months before pandemic onset at an urban safety net hospital in the US. Prepandemic data was extracted from the electronic medical record, and surveys were administered at three time points between August 2020 and July 2021. We measured mental health symptoms with the Pediatric Symptom Checklist-17, social risks (e.g., food and housing insecurity) with the THRIVE questionnaire, and school modality (in-person, hybrid, remote).

**Results:**

Compared to pre-pandemic, children had significantly higher PSC-17 total scores (overall mental health symptoms) and THRIVE total scores (total burden of social risks) at all three mid-pandemic waves. Using longitudinal mixed models accounting for time, social risks, and school modality, both social risks (B = 0.37, SE = 0.14, p < 0.01) and school modality were significantly associated with PSC-17 scores (B = − 1.95, SE = 0.63, p < 0.01). Children attending in-person school had fewer mental health symptoms than those attending remote or hybrid school.

**Conclusion:**

Mental health symptoms and social risks remained significantly higher fifteen months after the onset of the COVID-19 pandemic compared to prepandemic. In-person attendance at school appeared protective against persistently elevated mental health symptoms.

**Supplementary Information:**

The online version contains supplementary material available at 10.1186/s13034-024-00712-4.

## Background

Studies have documented the detrimental impact of the COVID-19 pandemic on pediatric mental health, exacerbating an existing downward trend and worsening barriers to accessing timely treatment [1–9]. In the US, longitudinal data from the 2016 through 2020 National Surveys of Children’s Health indicated increases in youth anxiety and depression that began before the pandemic; increases in anxiety, depression, and behavioral problems during the pandemic; and decreased access to both mental health and preventive medical care during the pandemic [3]. The COVID-19 pandemic introduced new acute stressors including social isolation, increased stress on caregivers, increased screen time, and online school [2]. A global meta-analysis of child mental health found that anxiety and depression rates doubled during the pandemic [1]. As a result, in October 2021 the American Academy of Pediatrics (AAP), the American Academy of Child and Adolescent Psychiatry (AACAP), and the Children’s Hospital Association (CHA) together declared a national emergency for youth mental health [10]. Shortly thereafter, the US Surgeon General issued an advisory highlighting the alarming rate of pre-pandemic youth mental health problems that were exacerbated by pandemic-related stressors [11]. The advisory highlighted the disproportionate impact of COVID-19 on already vulnerable communities, including youth living in low-income households, racial and ethnic minoritized youth, and youth in the child welfare or juvenile justice system [11].

The impact of COVID-19 on school-age children is of particular concern given the disruptions of critical developmental milestones (e.g., academics and socialization) and the influence of preadolescent mental health problems on future symptoms and functioning [12, 13]. Despite this, few published studies describe the longitudinal impact of COVID-19 and its related stressors on the mental health of children in the US; even fewer study preadolescent children or the vulnerable populations highlighted by the Surgeon General [11]. Existing studies are primarily cross-sectional, examine adolescent mental health only, do not include pre-pandemic comparison data, do not evaluate the impact of increased financial hardship and change in social risks (e.g., food or housing insecurity), and/or do not focus on vulnerable populations [4–6, 9, 14]. Understanding the trajectory of child mental health during the first two years of the pandemic, including the relationship between symptoms and other pandemic-related stressors, has important practice and policy implications. Documenting any persistent impact of the pandemic on preadolescent, school-aged children is particularly critical, as this generation may need support throughout childhood and adolescence.

Our objective was to better understand the progression of mental health symptoms during the pandemic among school-aged children and the relationship between mental health and social risks and other mid-pandemic stressors. To achieve this goal, we conducted a longitudinal study, including pre-pandemic data, to evaluate the impact of social risks and remote school on mental health among urban, minoritized (predominantly Black and Latino/a/x/e) children aged 6–11 years old. We published initial results from this cohort after one wave of data collection during the first year of the pandemic, documenting significant increases in internalizing (depression and anxiety) problems and social risks [7]. Child mental health problems were associated with lower school assignment completion, caregiver mental health, and increased screen time [7]. The current study includes two additional waves of data collection during the second year of the COVID-19 pandemic to assess the ongoing impact of pandemic-related stressors on child mental health. We examined trajectories in mental health symptoms over time and evaluated correlates of mental health, including social risks (e.g., food insecurity) and school modality. We hypothesized that social risks and school modality would be associated with worse child mental health across time.

## Methods

### Setting and recruitment

We conducted a longitudinal cohort study of children to evaluate the trajectory of child mental health symptoms during the COVID-19 pandemic at an urban, hospital-based, academic pediatric primary care practice at Boston Medical Center (BMC). BMC is the largest safety-net hospital in New England and serves a racially and ethnically diverse and primarily publicly insured patient population. Study procedures were approved by the Boston University Medical Campus Institutional Review Board. The study design and recruitment process have also been previously described. [7]

We identified eligible children using the electronic health record (EHR). Children were eligible if they were aged 6–11 years old at the time of recruitment; the preferred language documented in the EHR was English, Spanish, or Haitian Creole; and mental health screening results for the Pediatric Symptom Checklist (PSC-17) were documented from a well child visit at BMC in the 6 months prior to the onset of the novel coronavirus pandemic in Boston (between September 1, 2019, and March 1, 2020). Trained research staff contacted legal guardians (i.e., “caregivers”) of eligible children by phone in their preferred language to invite them to participate in the study. Informed consent procedures were conducted via phone and documentation of consent was collected electronically using REDCap.

Using the EHR, we extracted item-level results on the pre-pandemic PSC-17 and THRIVE (social risks) assessments, both of which are routinely completed at well child visits in pediatric primary care at BMC. Caregivers were invited to complete online surveys, including these two measures, at three time points during the mid-pandemic study period (August 2020-July 2021). Surveys were sent to consenting caregivers via email or text. Caregivers could also choose to complete surveys by telephone with research staff. Participants completed their first mid-pandemic surveys (“Wave 1”) between August 2020 and January 2021. To track the longitudinal impact of the pandemic on child mental health and social risks, two subsequent surveys were sent to participants 3 months (“Wave 2’) and 6 months (“Wave 3”) after they completed the first survey. Data collection concluded in July 2021. Caregivers received a $20 gift card for each completed survey.

## Measures

### Mental health symptoms

The PSC-17 is a validated, caregiver-report questionnaire available in many languages that assesses 17 emotional and behavioral health symptoms on a Likert scale (never = 0, sometimes = 1, or often = 2) [15–18]. The sum of all items yields a total score representing the severity of overall emotional and behavioral symptoms. Higher scores indicate more severe symptoms, and a total score of 15 or greater (referred to as “at-risk”) indicates the possibility of clinically concerning symptoms and need for further evaluation. An extensive body of research on the PSC-17 has demonstrated good reliability and validity for detecting children at high clinical risk [15–18]. In our sample, the total scores demonstrated adequate internal consistency, with Cronbach’s α coefficients ranging from 0.88 to 0.90.

### Social risks

All pediatric primary care patients at BMC are screened for social risks with the THRIVE survey that was developed at BMC [19] by adapting the WE CARE (Well Child Care, Evaluation, Community Resources, Advocacy, Referral, Education) social needs screening and referral program [20]. This caregiver-report survey instrument assesses for eight risks: housing, food, employment, and financial insecurity, as well as difficulty obtaining or accessing medication, transportation to medical appointments, or dependent care. Like WE CARE, THRIVE screens for both risks (e.g., “Do you have trouble getting transportation to medical appointments?”) and needs (e.g. “Do you need help connecting to resources?”). As we have done before with WE CARE, we included the number of *risks*, not needs, as the total THRIVE score for this analysis. Individual social risks were calculated by assigning a value of “1” to a risk, and “0” to no risk. We calculated a total THRIVE score (ranging from 0 to 8) to indicate the total number of caregiver-reported risks at each time point [21]. Two of the risks in THRIVE are assessed with more than one question (food and housing). Food insecurity is assessed with three questions that comprise the validated “hunger vital sign” measure [22]. Any answer other than “No” or “Never True” on any of these questions indicates food insecurity and was coded as a “1.” We followed this same pattern for scoring the two housing questions.

### School modality

Beginning at Wave 2, our survey included a question about school modality (remote/hybrid/in-person; this was because Boston Public Schools began returning some students to classrooms at the beginning of Wave 2 (mid-January 2021), after operating entirely remotely from March 2020 until that point [23]. For our analyses, we extrapolated that all children in Wave 1 (August 2020 to early January 2021) were attending school remotely, and that before the pandemic all children were attending school in-person.

### Open-ended survey questions

We collected written qualitative data with the following three optional, open-ended survey items at mid-pandemic data collection points: (1) please tell us anything else you think is important about how coronavirus has impacted your child's health or well-being (asked at all 3 mid-pandemic waves); (2) please tell us what things have been *most difficult* about school during the COVID-19 pandemic (Waves 2 and 3); and (3) please tell us what things have been *most rewarding* about school during the COVID-19 pandemic (Waves 2 and 3).

## Analysis

### Quantitative analysis

Quantitative analysis was conducted in Stata 17 [24]. For continuous variables, we evaluated normality using both the Wilk-Shapiro test and the test statistics for skewness and kurtosis [25, 26]. Variables were considered to violate the normality assumption if both the Wilk-Shapiro test and at least one of the test statistics for skewness and kurtosis were considered non-normal. To address non-normality, we conducted Wilcoxon signed-rank tests in addition to paired sample t-tests to compare PSC-17 total scores and THRIVE scores at each mid-pandemic wave with the pre-pandemic period. We used two-sample tests of proportion to compare categorical variables (PSC-17 at-risk scores and individual social risks) at each mid-pandemic wave compared to pre-pandemic. Cohen's d was used as an effect size for t-tests (small = 0.2, medium = 0.5, large = 0.8). 

We used linear mixed regression models (LMMs) with maximum likelihood estimation to estimate how social risks and school modality (remote/hybrid/in-person) were associated with mental health symptoms across time [27, 28]. LMMs offer more flexibility than repeated measures ANOVA in accounting for unbalanced and missing data and the inclusion of time-varying covariates [27]. A robust maximum likelihood estimator with adjustment for standard errors was used to account for non-normality in the data.

After graphing PSC total scores across the four time points, we fit a series of models to determine the relationship between time and mental health symptoms. We used the likelihood ratio χ [2] test (LRT) for nested models and the Akaike & Bayesian Information Criteria (AIC and BIC) to evaluate model fit [27–29]. After selecting the model that best represented the relationship between time and mental health symptoms, we estimated three additional models. One model included a variable for social risks (Model 1), another included variables for school modality (Model 2), and a final model included variables for both social risks and school modality (Model 3). In all quantitative analyses, the threshold for statistical significance was set at p = 0.05.

### Qualitative analysis

We analyzed open-ended survey responses during all mid-pandemic waves using content analysis [30, 31]. Two researchers (DYK and RD) examined the responses to the three separate open-ended questions and developed a group of codes for each item that characterized the responses from participants. An inductive approach was utilized to account for emergent themes that were not reflected by pre-existing codes [31]. Both researchers then coded the remaining data for each item and met to review and discuss discrepancies. Finally, the entire research team reviewed and refined the codes, collapsing similar codes when appropriate. We calculated frequencies for each final code and compared answers to all 3 items within each wave as well as answers to each item across waves. Qualitative findings were integrated to identify trends observed in our quantitative findings as well as new or contrasting themes.

## Results

### Sample characteristics

We identified 910 eligible children in the EHR and reached 508 of their caregivers by phone (55.6%). Of those, 277 caregivers consented to participate (54.5%) and 172 caregivers (33.9%) completed the survey for at least the first wave of mid-pandemic data collection. There were no significant differences between the eligible sample and enrolled sample in terms of age, race/ethnicity, preferred language, insurance status or pre-pandemic mental health symptoms; however, male children represented a greater proportion (56.4%) of the final sample than the sample of eligible children (48.0%) [7]. Of the 172 caregivers who participated in the first wave of data collection, 147 (85.5%) participated in the second wave (i.e., answered 3-month surveys) and 136 (79.1%) participated in the third wave (i.e., answered 6-month surveys).

Sample demographic information is presented in Table 1. Children in our sample had an average age of 8.5 years (SD = 1.8) during their initial well visit (prior to the pandemic). There were no significant differences in terms of age, sex, race/ethnicity, language, or pre-pandemic mental health symptoms between participants who completed Wave 1, Wave 2, and Wave 3 of the survey.

**Table 1:** Sample Demographics (N = 172)

	Frequency	%
Child age- *M* (*SD*)	8.5 (1.8)	–
Child sex		
Male	97	56.4
Child race/ethnicity		
Non-Latino Black	94	54.7
Latino	48	27.9
White	9	5.2
Other	3	1.7
Unknown	18	10.5
Caregiver preferred language		
English	133	77.3
Haitian Creole	29	16.9
Spanish	10	5.8
Child insurance		
Public	124	72.1
Commercial	48	27.9

### Mental health symptoms

As shown in Table 2, higher levels of child mental health symptoms were reported at all mid-pandemic waves compared with the pre-pandemic baseline. Average PSC-17 total scores were significantly higher at Wave 1 (*M* = 7.99, *SD* = 6.41, *t*_151_ = -6.03, *p* < 0.001, *d* = 0.50), Wave 2 (*M* = 7.70, *SD* = 5.97, *t*_131_ = − 5.76, *p* < 0.001, *d* = 0.50), and Wave 3 (*M* = 7.03, *SD* = 6.03, *t*_118_ = − 3.91, *p* < 0.001, *d* = 0.36), than during the pre-pandemic period (*M* = 5.62, *SD* = 5.80). Significantly more children had an at-risk score on the PSC-17 total score at Wave 1 than during the pre-pandemic period (17.6% vs. 7.9%, *z* = − 2.61, *p* < 0.01); categorical at-risk total scores at Wave 2 and Wave 3 data collection were not significantly different from scores during the pre-pandemic period.

**Table 2:** Longitudinal Trajectory of Mental Health Symptoms, Social Risks, and School Modality from August 2020 to July 2021 during the COVID-19 Pandemic Compared to the 6 months pre-pandemic (September 2019 – February 2020)

	Pre-pandemic (start Sep-2020)	Wave 1^1^ (start Aug-2020)	Wave 2^1^ (start Dec-2020)	Wave 3^1^ (start Mar-2020)
Child emotional and behavioral symptoms				
PSC-17 total score				
Median score (IQR)	4 (1, 9)	6 (3, 13)***	6 (3, 11)***	5 (2, 11)***
Mean score (SD)	5.62 (5.80)	7.99 (6.41)***	7.70 (5.97)***	7.03 (6.03)***
% positive	7.9%	17.6%**	14.7%	11.9%
Social risks (THRIVE)				
Median score (IQR)	0 (0, 5)	2 (0, 6)***	1 (0, 6)***	1 (0, 6)***
Mean score (SD)	0.87 (1.46)	1.76 (1.87)***	1.67 (1.78)***	1.48 (1.69)***
Specific risks				
% housing insecure	2.9%	11.5%**	12.5%**	8.8%*
% food insecure	15.7%	50.0%***	40.1%***	34.3%***
% difficulty affording medications	5.7%	9.0%	8.3%	8.1%
% difficulty with transportation	12.1%	13.0%	9.5%	11.1%
% difficulty paying bills	15.8%	36.6%***	33.3%**	30.6%
% difficulty with dependent care	0.8%	10.2%**	7.5%**	5.9%*
% unemployed	13.9%	23.2%*	25.3%*	21.6%
% interested in more education	20.3%	38.5%***	33.6%*	29.4%
School modality				
Remote only	0%^2^	100%^3^	63.9%	43.6%
Hybrid	0%^2^	0%^3^	25.7%	24.8%
In-person only	100%^2^	0%^3^	10.4%	31.6%

PSC-17 = Pediatric Symptom Checklist (17-item version), IQR = interquartile range, SD = Standard Deviation

^1^All comparisons are between pre-pandemic and indicated wave of data collection; *p < .05, **p < .01, ***p < .001

^2^Not measured (based on pre-pandemic norms)

^3^Not measured (local public school district fully remote at this time)

### Social risks

Total scores on the THRIVE screener and the proportions of each reported social risk factor for each time point are shown in Table 2. Average total THRIVE scores were significantly higher at Wave 1 (*M* = 1.76, *SD* = 1.87, *t*_103_ = − 5.93, *p* < 0.001, *d* = 0.59), Wave 2 (*M* = 1.67, *SD* = 1.78, *t*_90_ = − 4.73, *p* < 0.001, *d* = 0.50), and Wave 3 (*M* = 1.48, *SD* = 1.69, *t*_85_ = − 3.62, *p* < 0.001, *d* = 0.39), than during the pre-pandemic period (*M* = 0.87, *SD* = 1.46). The proportion of caregivers reporting housing insecurity, food insecurity, difficulty paying bills, and difficulty with dependent care was significantly higher at each wave of mid-pandemic data collection than during the pre-pandemic period.

### School modality

The modality by which children attended school at Waves 2 and 3 of data collection is shown in Table 2. At Wave 2, almost two-thirds of children were participating in remote school, approximately one-quarter were participating in a hybrid school model, and 10.4% were attending school fully in person. At Wave 3, less than half of children were participating in remote school, approximately one-quarter were participating in a hybrid school model, and almost one-third were attending school fully in person.

### Longitudinal analysis

**Fig. 1:**
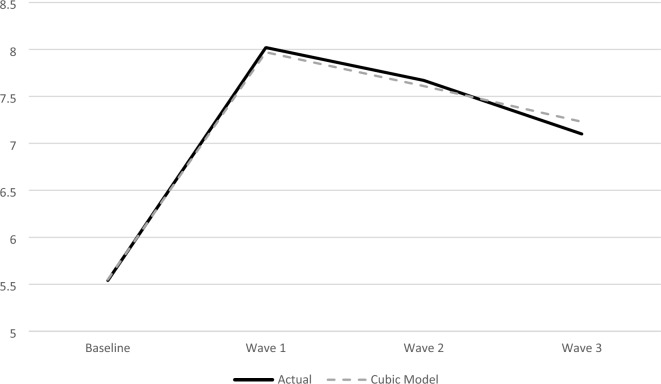
PSC Total Score over Time

To further examine the relationship between time and mental health symptoms, we manually graphed this relationship and estimated a series of LMMs We tested whether the relationship between time and mental health symptoms was best represented by a linear, quadratic, or cubic function. Results from these three models, including fit statistics, are reported in Additional file 1: Table S1. We also graphed each function alongside the raw data. LRTs indicated that the cubic model was significantly better than the linear (Δχ^2^ = 27.08, Δdf = 2) model but not the quadratic model (Δχ^2^ = 0.08, Δdf = 1). AIC values favored the cubic model and BIC values favored the quadratic. Based on visual examination, LRTs, and the information criteria, we determined that the cubic model best represented the data. Figure 1 shows both the cubic function alongside the study data. Additional file 1: Figure S1 shows the study data with the linear, quadratic, and cubic functions.

We then estimated three additional multivariable longitudinal models to examine the relationship between social risks, school modality, and mental health symptoms. The full results for these models, including fit statistics, are shown in Additional file 1: Table S2. Model 1 included THRIVE scores, Model 2 included school modality, and Model 3 included both THRIVE scores and school modality. Results, including fit statistics for each model, are included in Additional file 1: Table S3. THRIVE scores (indicating number of social risks) were significantly and positively associated with PSC total scores in both Model 1 (B = 0.40, SE = 0.14, *p* < 0.01) and Model 3 (B = 0.37, SE = 0.14, *p* < 0.01). Youth who attended school in person had significantly lower PSC total scores (indicating fewer mental health symptoms) than youth attending remote school only, in both Model 2 (B = − 1.95, SE = 0.65, *p* < 0.01) and Model 3 (B = − 1.94, SE = 0.63, *p* < 0.01). Youth who attended school in person had significantly lower PSC total scores than youth attending hybrid school in Model 2 (B = − 1.58, SE = 0.69, *p* < 0.05) and Model 3 (B = − 1.36, SE = 0.66, *p* < 0.05). There were no significant differences in PSC-17 total score between youth attending hybrid and remote school in Model 2 or 3. In both models accounting for school modality, the relationship between time and PSC-17 total score attenuated and did not reach significance.

We estimated a final model including THRIVE scores, school modality, and sociodemographic variables (child age, child sex, child race/ethnicity, caregiver preferred language, and child insurance). As in the earlier models, THRIVE scores were significantly and positively associated with child mental health symptoms (B = 0.39, SE = 0.14, *p* < 0.01). Youth who attended school in person had significantly lower PSC-17 total scores than youth attending remote school (B = − 1.69, SE = 0.68, *p* < 0.05). There were no significant differences in mental health symptoms between youth attending hybrid and remote school. Full results, including fit statistics and coefficients for sociodemographic variables are included in Additional file 1: Table S3.

## Qualitative content analysis

### Impact of COVID-19 on child’s health or well-being

Table 3 shows the qualitative content analysis results for each open-ended survey item at each wave. Item 1 (regarding the impact of COVID-19 on child’s health or wellbeing) was completed by 49 caregivers in Wave 1 and by 78 and 61 caregivers in Waves 2 and 3, respectively (Table 3a). At every wave the most common caregiver response was that their child’s mental or physical health had suffered (35%, 42%, and 41% of respondents in Waves 1, 2, and 3 respectively). Concerns about the child being unable to leave the home were also common, but were highest in Wave 1 (35%) and decreased over time (26% in Wave 2 and 23% in Wave 3). Concerns about social isolation were also common but were highest in Wave 3 (41% at Wave 3 compared to 27% at Wave 1 and 26% at Wave 2). COVID-19-related safety concerns were mentioned frequently in Wave 1 (27%) and least often in Wave 2 (14%). Educational problems were most commonly reported in Wave 1 (20% vs. 3% in Wave 2 and 5% in Wave 3).

**Table 3:** Content analysis of caregiver answers to open-ended survey items

**a**.“Please tell us anything else you think is important about how coronavirus has impacted your child’s health or wellbeing.”			
	Wave 1 N (%)	Wave 2 N (%)	Wave 3 N (%)
Total respondents	49 (100)	78 (100)	61 (100)
Child health detriment (mental, behavioral, or physical)	17 (35)	33 (42)	25 (41)
Child unable to leave home	17 (35)	20 (26)	14 (23)
Social difficulties including isolation	13 (27)	20 (26)	25 (41)
Concerns about safety	13 (27)	11 (14)	14 (23)
Educational problems	10 (20)	2 (3)	3 (5)
General difficulty	7 (14)	4 (5)	2 (3)
No impact	3 (6)	6 (8)	6 (10)
Lack of resources or unmet social needs	2 (4)	3 (4)	0 (0)
Caregiver mental health	1 (2)	4 (5)	0 (0)
Difficulty accessing healthcare for child or caregiver	0 (0)	4 (5)	1 (2)

**b**. “Please tell us what things have been MOST DIFFICULT about school during the COVID-19 Pandemic.”		
	Wave 2 N (%)	Wave 3 N (%)
Total respondents	129 (100)	110 (100)
Remote school difficulties	85 (66)	49 (45)
Social difficulties including isolation	46 (36)	22 (20)
Child mental health/behavioral difficulties	17 (13)	7 (6)
No problems	11 (9)	7 (6)
COVID-19 safety concerns/difficulty with COVID hygiene	10 (8)	14 (13)
Lack of resources or unmet social needs	10 (8)	8 (7)
Difficulty with hybrid school	5 (4)	2 (2)
Positive school experience	4 (3)	1 (1)

**c.** “Please tell us what things have been MOST REWARDING about school during the COVID-19 Pandemic.”		
	Wave 2 N (%)	Wave 3 N (%)
Total respondents	115 (100)	112 (100)
Better family relationship/Increase in time spent with family	24 (21)	26 (23)
Grateful for child-peer social interaction	7 (6)	3 (3)
Child doing better emotionally/physically/educationally	20 (17)	23 (21)
Remote school	21 (18)	19 (17)
Hybrid/in-person school	5 (4)	7 (6)
School effort/accommodation for children	17 (15)	9 (8)
Increased caregiver engagement in child’s education/ability to watch child develop	10 (9)	15 (13)
Nothing	21 (18)	11 (10)

### Most difficult part of school during COVID-19

Item 2 (Table 3b) prompted caregivers to share what was most difficult about school during COVID-19. This item was included in the survey during mid-pandemic Waves 2 and 3 only; 129 caregivers in Wave 2 and 110 caregivers in Wave 3 responded. The most common response was that remote school was most difficult (66% in Wave 2 and 45% in Wave 3). Social difficulties were the next most common response (36% in Wave 2 and 20% in Wave 3). Difficulty related to child mental health or behavior was more commonly reported in Wave 2 than Wave 3 (13% vs. 6% in Wave 3). COVID-19-related hygiene or safety concerns were more commonly reported in Wave 3 than Wave 2 (13% in Wave 3 vs. 8% in Wave 2).

### Most rewarding part of school during COVID-19

Item 3 prompted caregivers to share what made school most rewarding during COVID-19. This item was included in the survey during mid-pandemic Waves 2 and 3 only, and 115 caregivers in Wave 2 and 112 caregivers in Wave 3 responded. Caregivers most commonly reported more quality family time and better family relationships (21% in Wave 2 and 23% in Wave 3). Some caregivers also reported that remote school was rewarding (18% in Wave 2 and 17% in Wave 3) and that their child was doing generally better than pre-pandemic (17% in Wave 2 and 21% in Wave 3).

## Discussion

Our study examines longitudinal mental health during the COVID-19 pandemic in a predominantly minoritized sample of urban elementary school children in the United States. Children in this sample had significant increases in emotional and behavioral symptoms—measured with PSC total scores—that persisted through the first fifteen months of the pandemic. Families also faced significantly more social risks at all waves during the pandemic than before. In longitudinal models accounting for social risks and school modality, social risks were associated with increased mental health symptoms. In-person school attendance was associated with improved mental health, as compared with both hybrid and remote school.

Our findings are consistent with other research showing a rise in child mental health problems related to the pandemic [1, 2, 5, 6, 8, 9, 32–36]. The findings that PSC total scores had not returned to normal levels by the end of our study period support calls for action to meet the increased demand for child mental health services and prevent more serious long-term consequences. [37, 38]

The persistence of mental health symptoms may be due to multiple factors, including ongoing school disruption and social isolation, reduced access to afterschool and enrichment programs, increased hospitalizations and death rates due to COVID-19 among communities of color [39], unmet social needs and socioeconomic concerns, and decreased social supports for families. In open-ended responses, caregivers noted persistent concerns about safety due to COVID-19, including during later waves when children returned to in-person school (which increased risk of exposure). Caregivers also shared concerns about social isolation, even after children had begun returing to in-person school.

Children and families in our sample faced significantly more social risks throughout the first 15 months of the pandemic than before, including housing insecurity, food insecurity, financial insecurity, and difficulty obtaining dependent care. Half of participants during Wave 1 reported food insecurity, which decreased to approximately one-third of participants by Wave 3 (as compared with approximately 15% prior to the pandemic). The high persistence of social risks in our sample is consistent with other research [9, 40]. In an analysis of longitudinal data from the Adolescent Brain Cognitive Development (ABCD) study, Xiao et al. [9] found that multiple social risks, including food insecurity, disproportionately impacted impacted racial and ethnic minority children. They further found that social risks were significantly associated with internalizing symptoms. Other chronic social stressors that disproportionately affect the mental and physical health of individuals from racial or ethnic minority groups, like structural racism, were exacerbated during the COVID-19 pandemic [41, 42], potentially impacting the relationship between social risks and mental health in this sample.

The persistence of social risks has critical policy and clinical implications. Attention to improving social supports and providing basic needs for families in the current phase of pandemic recovery should be an urgent public health priority. Pediatric primary care and mental health providers may be able to initiate support for families by using screening and referral programs (e.g., WE CARE [20]) and partnering with community organizations to connect families in need of care. In addition, policy advocates must work to ensure that public supports aimed at reducing social risks continue to support vulnerable populations that have been disproportionately impacted by the pandemic. Supporting families access to housing, food, and other basic needs should be a critical component of efforts to address the current pediatric mental health crisis.

Persistent remote school or hybrid school (as compared to in-person school) was also associated with child mental health in our sample. Caregivers of children attending school in person reported significantly fewer child mental health symptoms than those of children attending hybrid or remote school, even after accounting for social risks. Existing research documented racial disparities in persistent remote school attendance, with Black students more likely to attend remote school even after accounting for sociodemographic factors [43]. Thus, persistent remote schooling may have contributed to racial disparities in pandemic-related child mental health problems. In-person school attendance may be protective for child mental health for a number of reasons; schools address some social risks for students (e.g., access to free and reduced-price lunch), provide improved opportunities for peer socialization, and may promote increased physical activity compared to the home environment. Access to in-person school services may therefore be important for supporting child mental health. Enhancing school mental health supports may also play an important role in reducing child mental health problems.

An estimated 230,000 children nationwide have not returned to school since the onset of COVID-19 and are at high risk for mental health difficulties [44]. Efforts to re-engage these children and families should be an important public health effort. Primary care clinicians may help identify these children at office visits, but partnerships with educational and mental health professionals may also be required to to promote engagement with schools or other in-person activities. Children who remained in remote or hybrid school for an extended period of time may also be at higher risk for mental health problems; additional research is needed to understand how features of the pandemic, such as remote schooling and increased screen time, may impact the long term mental health of children.

Our study has some limitations. Our sample was limited to caregivers of pediatric primary care patients at one urban safety-net hospital, and may not generalize to other populations. The survey was only available in English, Spanish, or Haitian Creole. The longitudinal analysis was limited to variables measured both pre- and mid-pandemic, precluding the inclusion of variables such as screen time, caregiver mental health, and experiences of discrimination. Although using a THRIVE total score as an indicator of social risks has not been validated, we previously used this method in a study with the WE CARE screener (which was used to develop THRIVE) [21]. We did not collect school modality during the first wave of data collection (August 2020- January 2021) since all Boston Public Schools and most Massachusetts schools were exclusively remote from March 2020 to mid-January 2021 [23]. In addition, BMC’s pediatric patient population includes primarily publicly insured children living in Boston [45]. Therefore, for the purposes of longitudinal modeling, we felt comfortable assigning all children a value of ‘in-person only’ for the pre-pandemic time period and a value of ‘remote school only’ for Wave 1. Although it is possible that some students were attending private schools with hybrid or in-person options during the first wave of data collection or had been home schooled prior to and/or during the pandemic, we are confident this is a small number of children. All survey research involves the risk of non-response bias; we believe this risk was minimized through two important factors: (1) children in our sample did not differ significantly in baseline mental health symptoms from the larger population of pediatric primary care patients at our site; and (2) we had over 75% retention of participants across waves of data collection. Our study also relied on caregiver report of child mental health symptoms. It is possible that factors such as increased time spent with children during periods of remote schooling may have influenced caregiver perceptions of child symptomatology. Finally, our hypothesis driven study involved multiple comparisons; given our small sample size, we did not adjust our p-value because of concern about Type II error.

Our study also has strengths. The mixed methods design allowed us to highlight caregiver reported concerns that were not assessed in quantitative survey questions. To reduce barriers to participation, the survey was available in three languages and families could participate by smart phone, computer, or telephone. Our research protocols also allowed us to support caregivers who reported social needs during data collection. We connected caregivers with primary care teams and federal, state, and community-level resources to help alleviate stressors such as food insecurity, housing insecurity, and difficulty with dependent care as part of the study protocol.

## Conclusion

School-aged racially and ethnically minoritized urban children had persistently elevated mental health symptoms at three time points during the COVID-19 pandemic. Fifteen months after the onset of the pandemic, social risks, including food and housing insecurity, remained worse than before the pandemic. Social risks were associated with increased mental health symptoms across time. Full return to in-person school was associated with decreased mental health symptoms, as compared with both remote and hybrid school. Ensuring that children return to school in-person and that families’ basic needs are met may be important steps in addressing the current child mental health crisis. It is essential to provide school-aged children with additional resources for treatment, both within and beyond the school environment, to protect them from the ongoing mental health impact of COVID-19.

### Supplementary Information

**Additional file 1:** **Figure S1.** Model Selection: PSC-17 Total Problems Score over Time. **Table S1.** Linear Mixed Models: The Relationship between Time and PSC-17 Total Problems Score. **Table S2.** Linear Mixed Models: The Relationship between Time, Social Risks, and School Modality with PSC-17 Total Problems Score. **Table S3**. Final Model: The Relationship between Time, Social Risks (measured with THRIVE), School Modality, and Sociodemographics with Mental Health Symptoms (measured with the PSC-17 Total Problems Score).

## Data Availability

The dataset generated and analyzed for survey responses from participants of the study are not publicly available due to containing potentially identifiable information, but are available from the corresponding author on reasonable request. The datasets used during the current study to support the finding that all participants of the study were attending remote school during the first wave of longidudinal data collection are available in the Center for Open Science Repository, https://osf.io/zeqrj/.[23]
