# Supplementary material for: Trajectories and correlates of mental health among urban, school-age children during the COVID-19 pandemic: a longitudinal study
Source: Child Adolesc Psychiatry Ment Health. 2024 Mar 14;18:32. doi: 10.1186/s13034-024-00712-4 (PMC10941406; doi:10.1186/s13034-024-00712-4)
Supplement: Supplementary file 1 — Additional file 1: Figure S1. Model Selection: PSC-17 Total Problems Score over Time. Table S1. Linear Mixed Models: The Relationship between Time and PSC-17 Total Problems Score. Table S2. Linear Mixed Models: The Relationship between Time, Social Risks, and School Modality with PSC-17 Total Problems Score. Table S3. Final Model: The Relationship between Time, Social Risks (measured with THRIVE), School Modality, and Sociodemographics with Mental Health Symptoms (measured with the PSC-17 Total Problems Score). [file 13034_2024_712_MOESM1_ESM.docx]

**Figure S1.** Model Selection: PSC-17 Total Problems Score over Time

**Table S1.** Linear Mixed Models: The Relationship between Time and PSC-17 Total Problems Score

|  | **Linear** | | **Quadratic** | | **Cubic** | |
| --- | --- | --- | --- | --- | --- | --- |
|  | Coefficient | Standard Error | Coefficient | Standard Error | Coefficient | Standard Error |
| Time |  |  |  |  |  |  |
| Time | 0.50*** | 0.13 | 2.61*** | 0.41 | 4.56*** | 0.98 |
| Time^2^ |  |  | -0.73*** | 0.13 | -2.65** | 0.81 |
| Time^3^ |  |  |  |  | 0.43* | 0.17 |
|  |  |  |  |  |  |  |
| Model Fit |  |  |  |  |  |  |
| ICC | 0.73 | | 0.74 | | 0.74 | |
| Likelihood Ratio Test | 15.31*** | | 42.31*** | | 42.39*** | |
| AIC | 3279.92 | | 3251.33 | | 3248.25 | |
| BIC | 3297.28 | | 3273.02 | | 3274.28 | |

**p*<.05, ***p*<.01, ****p*<.001

ICC= intraclass correlation coefficient

AIC=Akaike information criterion

BIC=Bayesian information criterion

PSC-17=Pediatric Symptom Checklist (17-item version)

**Table S2.** Linear Mixed Models: The Relationship between Time, Social Risks, and School Modality with PSC-17 Total Problems Score

|  | **Model 1** | | **Model 2** | | **Model 3** | |
| --- | --- | --- | --- | --- | --- | --- |
|  | Coefficient | Standard Error | Coefficient | Standard Error | Coefficient | Standard Error |
| Time |  |  |  |  |  |  |
| Time | 3.63*** | 1.09 | 0.77 | 1.44 | -0.03 | 1.50 |
| Time^2^ | -2.12* | 0.86 | -0.41 | 1.05 | 0.09 | 1.08 |
| Time^3^ | 0.35 | 0.18 | 0.06 | 0.21 | -0.03 | 0.21 |
| THRIVE | 0.40** | 0.14 |  |  | 0.37** | 0.13 |
| School Type^1^ |  |  |  |  |  |  |
| Hybrid |  |  | -0.36 | 0.48 | -0.58 | 0.47 |
| In-Person Only |  |  | -1.95** | 0.58 | -1.94** | 0.62 |
|  |  |  |  |  |  |  |
| Model Fit |  | |  |  |  | |
| ICC | 0.78 | | 0.75 | | 0.79 | |
| Likelihood Ratio Test | 42.34*** | | 49.83*** | | 54.39*** | |
| AIC | 2735.81 | | 3215.77 | | 2703.37 | |
| BIC | 2765.05 | | 3250.41 | | 2740.88 | |

**p*<.05, ***p*<.01, ****p*<.001

^1^Reference Group is Remote School

ICC= intraclass correlation coefficient

AIC=Akaike information criterion

BIC=Bayesian information criterion

PSC-17=Pediatric Symptom Checklist (17-item version)

**Table S3.** Final Model: The Relationship between Time, Social Risks (measured with THRIVE), School Modality, and Sociodemographics with Mental Health Symptoms (measured with the PSC-17 Total Problems Score)

|  | **Model 1** | |
| --- | --- | --- |
|  | Coefficient | Standard Error |
| Time |  |  |
| Time | 0.42 | 1.60 |
| Time^2^ | -0.28 | 1.12 |
| Time^3^ | 0.05 | 0.22 |
| THRIVE | 0.39** | 0.14 |
| School Type^1^ |  |  |
| Hybrid | -0.65 | 0.49 |
| In-Person Only | -1.69* | 0.67 |
| Child Age | -0.41 | 0.25 |
| Female^2^ | -1.23 | 0.89 |
| Race/Ethnicity^3^ |  |  |
| Hispanic | 2.11 | 1.22 |
| Non-Hispanic White | 3.33* | 1.63 |
| Other | -3.13* | 1.36 |
| Caregiver Preferred Language^4^ |  |  |
| Haitian Creole | -2.09 | 1.40 |
| Spanish | -2.99 | 1.90 |
| Private Insurance^5^ | -1.46 | 0.88 |
|  |  |  |
| Model Fit |  | |
| ICC | 0.79 | |
| Likelihood Ratio Test | 81.71*** | |
| AIC | 2305.07 | |
| BIC | 2373.39 | |

**p*<.05, ***p*<.01, ****p*<.001

^1-5^Reference Groups are Remote School, Male, Non-Hispanic Black, English, and Public Insurance

ICC= intraclass correlation coefficient

AIC=Akaike information criterion

BIC=Bayesian information criterion

PSC-17=Pediatric Symptom Checklist (17-item version)
